# Faster-growing parasites threaten host populations via patch-level population dynamics and higher virulence; a case study in *Varroa* mites (Mesostigmata: Varroidae) and honey bees (Hymenoptera: Apidae)

**DOI:** 10.1093/jisesa/ieae049

**Published:** 2024-05-28

**Authors:** Lewis J Bartlett, Michael Boots, Berry J Brosi, Keith S Delaplane, Travis L Dynes, Jacobus C de Roode

**Affiliations:** Center for the Ecology of Infectious Diseases, Odum School of Ecology, University of Georgia, Athens, GA, USA; Department of Entomology, University of Georgia, Athens, GA, USA; Department of Integrative Biology, UC Berkeley, Berkeley, CA, USA; Biosciences, University of Exeter, Penryn Campus, Falmouth, UK; Department of Biology, University of Washington, Seattle, WA, USA; Department of Entomology, University of Georgia, Athens, GA, USA; Department of Environmental Sciences, Emory University, Atlanta, GA, USA; Department of Biology, Emory University, Atlanta, GA, USA

**Keywords:** *Varroa* mite, population growth, transmission, virulence

## Abstract

Honey bee parasites remain a critical challenge to management and conservation. Because managed honey bees are maintained in colonies kept in apiaries across landscapes, the study of honey bee parasites allows the investigation of spatial principles in parasite ecology and evolution. We used a controlled field experiment to study the relationship between population growth rate and virulence (colony survival) of the parasite *Varroa destructor* (Anderson and Trueman)*.* We used a nested design of 10 patches (apiaries) of 14 colonies to examine the spatial scale at which *Varroa* population growth matters for colony survival. We tracked *Varroa* population size and colony survival across a full year and found that *Varroa* populations that grow faster in their host colonies during the spring and summer led to larger *Varroa* populations across the whole apiary (patch) and higher rates of neighboring colony loss. Crucially, this increased colony loss risk manifested at the patch scale, with mortality risk being related to spatial adjacency to colonies with fast-growing *Varroa* strains rather than with *Varroa* growth rate in the colony itself. Thus, within-colony population growth predicts whole-apiary virulence, demonstrating the need to consider multiple scales when investigating parasite growth-virulence relationships.

## Introduction

An expansive body of work has documented a range of threats to embattled managed honey bees and, by extension or association, wild bees (Potts et al. 2016). One important determinant of honey bee and wider bee health is the arrival and subsequent evolution of novel or re-emerging parasites and pathogens (Manley et al. 2015). Both host evolution (van Alphen and Fernhout 2020) and parasite evolution (Ryabov et al. 2014, 2019) are current focusses of the honey bee health research arena, including continued emphasis on host and parasite phenotypes in determining the outcomes of infection or infestation (Brosi et al. 2017). Prior work has shown that strain differences in honey bee deformed wing virus lead to corresponding changes in both individual-level and colony-level virulence (McMahon et al. 2016) but meaningful phenotypic diversity relating to virulence of infestation has not, to our knowledge, been shown in the vector of this virus, the parasitic *Varroa* mite.

*Varroa* mites originated in Southeast Asia as an endemic parasite of the Asian honey bee, *Apis cerana*. They crossed into western honey bees, *Apis mellifera*, in the early 20th century and have since become a near-worldwide invasive parasite (Wilfert et al. 2016). They feed on the fat body and hemolymph of developing pupal and adult honey bees (Ramsey et al. 2019, Han et al. 2024), reproducing when brood is sealed in wax chambers. *Varroa* vector at least 1, and likely more, major viruses of honey bees (McMahon et al. 2018) and are a leading cause of managed honey bee losses in Europe and North America, where the industry expends substantial effort to control them (Hansen 2021, Jack and Ellis 2021).

The selection environment faced by *Varroa* varies based on the scale at which bees are kept (Dynes et al. 2019, 2020, Bartlett et al. 2021). Large, industrial, and migratory beekeeping operations present a very different ecology than small-scale, local, and geographically limited operations (Simone-Finstrom et al. 2016, Brosi et al. 2017). *Varroa* already show evolutionary adaptation against common control measures including chemical control (Guo et al. 2021, Millán-Leiva et al. 2021, Vlogiannitis et al. 2021). Thus, we presume they similarly experience selection on their growth rates (population increase with time), virulence (increased mortality), and transmission (spread between colonies), as other parasites do (Anderson and May 1982). However, *Varroa* growth-rate × virulence relationships are complicated by the hierarchical biological organization of their host: *Varroa* strains that are highly detrimental to individual bees reduce the size and strength of growing colonies, ultimately limiting total bee and parasite population size, and reducing opportunities for between-colony transmission.

*Varroa* remain challenging to control in part because of their recently evolved resistance to common synthetic miticides such as amitraz (Rinkevich 2020, Guo et al. 2021), tau-fluvalinate (Millán-Leiva et al. 2021), and coumaphos (Vlogiannitis et al. 2021), and the labor-intensiveness or biological limitations of alternatives, such as oxalic acid (Jack and Ellis 2021, Bartlett 2022, Berry et al. 2022). Current integrated pest management (IPM) guidelines recommend treating colonies only at some threshold of mite infestation, in part to reduce the rate of resistance evolution (Jack and Ellis 2021). However, it remains unclear whether the colony or the apiary is the appropriate “unit of treatment,” given the rates at which parasites can move between colonies (Nolan and Delaplane 2017). The hierarchical organization of host biology further complicates *Varroa* control. In any given apiary the colony with the largest *Varroa* population size may not be the one experiencing the highest per-capita rates of parasitism due to differences in colony size. Per-capita parasitism is typically used to establish treatment thresholds and is likely the trigger for facultative dispersal by the mite (Cervo et al. 2014), but this may potentially lead to the largest *Varroa* populations going untreated if the biggest colonies account for the highest absolute mite populations—despite having lower per-capita mite numbers.

Here, we used a replicated design in which we set up 10 circular apiaries of 14 colonies each. Initially, colonies were aggressively treated for *Varroa.* We then inoculated 2 colonies in each of the 10 apiaries using 10 different *Varroa* mite cohorts taken from different population sources. We then measured differences in *Varroa* population growth rates as well colony mortality, and asked at what level (colony versus apiary) mite population growth is related to colony survival.

## Methods

### Experimental Set-Up

We founded 140 new honey bee colonies in March 2015 from standardized 3lb packages purchased from a single beekeeping supplier and open-mated queens listed as Italian–Carniolan hybrids purchased from a second beekeeping supplier in Georgia, USA. All packages of bees were installed on single 10-frame Langstroth deeps with drawn comb with equal honey supplies and treated with an oxalic acid (Sigma-Aldrich, USA) sugar syrup solution upon purchase to reduce *Varroa* populations, and were then treated twice again with vaporized oxalic acid after colonies were established in hives to maintain low *Varroa* counts. Colonies were arranged in 10 circular apiaries of 14 colonies, each across the landscape of North Georgia, USA, with > 5 miles between each apiary. Colony entrances were outward-facing. Once colonies were fully established in April 2015, 2 colonies in each apiary (hereafter, “inoculated colonies”) were each inoculated with 400 *Varroa* sourced from research apiaries managed by our lab or local collaborating beekeepers. Inoculated colony pairs were diametrically opposite one another in the circular apiary. *Varroa* were sourced from research apiaries with diverse colony sources, including those from industrial migratory beekeeping regimes, local professional beekeepers, small-scale queen breeding regimes, and feral colonies from national wildlife areas in the region as discussed by Dynes et al. (2020) and Bartlett et al. (2021). Pairs of inoculated colonies within each research apiary received the same mix of donated mites, but no donor colony contributed mites to more than 1 single experimental apiary. *Varroa* were collected live from donor colonies using the “sugar shake” approach (Dietemann et al. 2013), whereby many thousands of adult honey bees were collected from the brood frames of donor colonies, covered in fine-ground sugar (powdered/icing/confectioners’ sugar), and agitated over a metal screen and collection tray whereby the white sugar and dislodged *Varroa* can pass through mesh apertures and fall to be collected. *Varroa* were then handled using fine-tip paint brushes and sorted into cohorts of 400 mites. Mites were introduced into colonies by distributing live mites across uncapped, pre-pupal brood cells in inoculated colonies. Using this approach, each experimental apiary was seeded with *Varroa* populations which we presume differ in genotype and phenotype. Following inoculation, all colonies were otherwise managed by apicultural technicians according to modified standard beekeeping practices, with the exceptions that no *Varroa* or parasite control was used, no brood or bees were moved between colonies, colonies were not combined, locations were not changed, and any action that might incidentally move mites between colonies was not taken. We refer to the 120 colonies that were not inoculated but shared an apiary with 2 inoculated colonies as “exposed” colonies hereafter.

### Data Collection

We take March 2015 to be “month 1.” We sampled mite population sizes in each colony using sticky-screen bottom boards, where screens were placed on bottom boards for 72 h to collect fallen/deceased *Varroa* mites, starting in month 2. Note this does not directly inform per-capita parasitism (mites per bee or per brood cell), but instead measures a proxy for total mite population size. We undertook sticky screen sampling in months 2, 4, 5, and 6, before bees entered their overwintering period in November (month 7). At the end of month 6, we assessed all colonies for size metrics (adult bees, brood coverage, and honey stores), by visually estimating the area of each frame covered by bees, capped brood, or capped honey. In month 7, as bees entered their overwintering phase, we measured per-capita mite parasitism by collecting approximately 300 adult bees from brood frames and dislodging phoretic mites by washing with 70% ethanol (“alcohol washes”), before counting the number of bees and mites. We followed colony survival from month 1 onward, for 12 months total, with inspections every month.

### Statistical Analysis

All analysis was undertaken in the open statistical software R v4.0.2 (R Core Team 2019). We estimated mite population growth rates in inoculated colonies in each apiary using a series of Poisson-distributed generalized linear mixed models (Bates et al. 2015) with the colony as a random effect, time as a fixed effect, and mite counts as the response variable. We use and interpret the coefficient found for the time fixed effect in this mite count response variable model as the “growth rate.” We similarly estimated mite population growth rates for typically exposed colonies in each apiary using the same modeling approach. We used typical Poisson-distributed generalized linear models (GLMs) to also estimate the total mite population growth rate in each apiary when mite counts were aggregated across all colonies in an apiary. At month 7, prior to overwintering, we used a Poisson-distributed GLM to test whether per-capita mite parasitism was significantly predicted by apiary and a binomial-distributed GLM to test if parasitism predicted overwintering mortality; we also used Pearson correlations to test whether there were significant correlations between per-capita mite parasitism and total mite population size and colony size. Finally, we assessed exposed-colony mortality from month 2 onward using a penalized nested right-censored frailty analysis with “frailtypack” (Rondeau et al. 2021), adjusting for our right-censored data and including apiary as a block effect. We make all necessary data and analysis available via a Zenodo archived repository [DOI: 10.5281/zenodo.10899687].

## Results

**Fig. 1.**
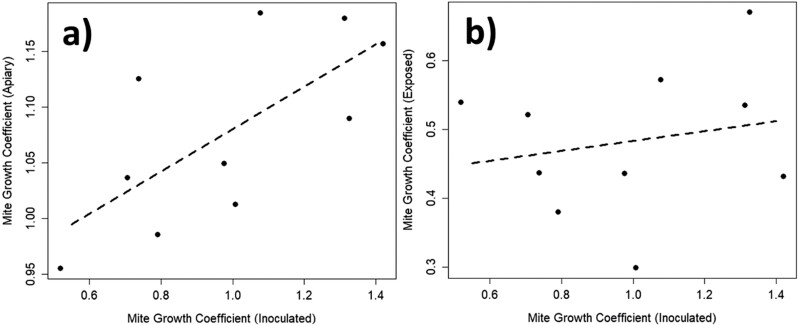
Correlations between estimated mite population growth rates in inoculated colonies and total mite population growth rate (panel a) or average inoculated colony mite growth rate (panel b) for each apiary (each data point corresponds to an apiary) over the 6 months following *Varroa* control treatments and subsequent inoculation. We found a significant, positive correlation between the speed at which mite populations grew in the inoculated colonies and the observed rate of total mite population growth in the entire corresponding apiary (*R*^2^ = 0.42, *F*_1,8_ = 7.58, *P* = 0.025, panel a). However, we found no significant correlation between the speed at which mite populations grew in the inoculated colonies and the observed rate of mite population growth in the rest of the exposed colonies in the same apiary (*F*_1,8_ = 0.34, *P* = 0.58, panel b).

**Fig. 2.**
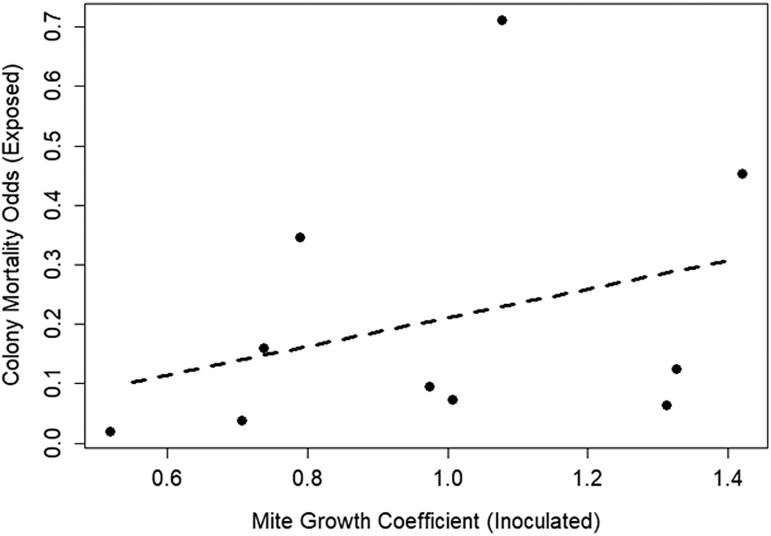
Simplified representation of the survival analysis undertaken, which found increased mortality rates in exposed colonies sharing an apiary with inoculated colonies harboring faster-growing *Varroa* populations (*P* = 0.030). Here, we show the estimated odds per timepoint of an exposed colony dying (as calculated by a binomial generalized linear model) correlated against the observed mite growth rate in inoculated colonies for each apiary. We highlight that the featured correlation is less sophisticated than the survival analysis undertaken and is intended as an illustration of the main finding only. The corresponding survival graph can be found in the Supplementary material (Supplementary Fig. S2).

As expected, mite populations in inoculated colonies grew rapidly during the first 6 months of the study prior to overwintering (Supplementary Fig. S1), and this led to mite population growth in exposed colonies as well (Supplementary Fig. S1). We found appreciable quantitative variation in mite population growth rates (see Supplementary Fig. S1). Upon examination, inoculated colony population growth rates did not correlate with mite growth rates in same-apiary exposed colonies (*F*_1,8_ = 0.34, *P* = 0.58; Fig. 1). However, there was a strong positive correlation between mite population growth rates in inoculated colonies and total mite population growth across each apiary (*R*^2^ = 0.42, *F*_1,8_ = 7.58, *P* = 0.025; Fig. 1). That is to say, total mite population growth in any given apiary was principally determined by the mite populations in the 2 inoculated colonies, not the 12 exposed colonies. Furthermore, mite population growth rates in inoculated colonies predicted the survival rates of exposed colonies in the same apiary (*P* = 0.030) where higher mite growth rates in inoculated colonies positively correlated with higher death rates in exposed colonies sharing that apiary (Fig. 2 and Supplementary Fig. S2). Prior to entering overwintering, exposed colony per-capita mite parasitism significantly differed between apiaries (χ^2^_9_ = 531.3, *P* < 0.001) and was positively correlated with total mite population size in each colony (*t*_81_ = 13.75, *P* < 0.001, Supplementary Fig. S3) but not with number of adult bees or brood area (*t*_81_ = −0.09, *P* = 0.927; *t*_81_ = 2.92, *P* = 0.771). In addition, exposed colony per-capita mite parasitism going into the overwintering period predicted colony survival into spring (χ^2^_81_ = 10.49, *P* = 0.001), where higher per-capita mite parasitism led to higher overwinter mortality rates.

## Discussion

We found evidence of a virulence × growth-rate relationship in *Varroa* using this controlled field experiment. Colonies exposed to neighboring *Varroa*-inoculated colonies were more likely to die according to the rate of growth of those neighboring *Varroa* populations. This appears to be driven by faster-growing in-colony *Varroa* populations leading to larger apiary-wide *Varroa* populations and subsequent large *Varroa* populations and higher per-capita *Varroa* parasitism when colonies contracted in size and entered their critical over-wintering period. Exposed colonies did not initially show correlations in their *Varroa* population growth rates with their inoculated neighbors (Fig. 1), suggesting this is not a simple effect of each apiary’s environment predisposing any given colony to faster *Varroa* growth, or that imported *Varroa* from the local landscape were determining survival. Instead, the presence of fast-growing *Varroa* populations in neighboring colonies leads to a higher whole-patch parasite burden across the apiary as colonies enter the critical late season and begin overwintering. Colloquially, beekeepers may understand this as “a few bad apples [colonies] spoil the bunch [apiary].”

This finding has parallels with other “patch-depletion” demonstrations of relationships between virulence and transmission/parasite growth rate, whereby more virulent, faster-growing parasites strains will “burn through” their local patch of resources much more quickly, which in turn may impact evolutionary outcomes based on the connectedness of a landscape. A substantial body of theory has explored the spatial constraint placed on virulence evolution due in part to these “local patch depletion” effects (Kamo and Boots 2006, Lion and Boots 2010), while empirically, Kerr et al. (2006) and Boots and Mealor (2007) showed such dynamics using a bacteria-phage system and a moth–virus system, respectively. These spatial dynamics may be critical for the selection of landscapes placed on *Varroa* by modern beekeeping (Dynes et al. 2019). However, industrial beekeeping operations rapidly replenish lost colonies within apiaries and provide easy transmission of parasite strains between colonies (Nolan and Delaplane 2017, Bartlett et al. 2019, Dynes et al. 2019), potentially selecting for more virulent honey bee parasites (Brosi et al. 2017, Dynes et al. 2020). Given our observed quantitative variation in virulence × growth-rate relationships, such selection is likely to occur in modern beekeeping operations.

From a management perspective, it is clear that the *Varroa* dynamics in neighboring colonies are important for predicting the mortality risk of colonies within a whole apiary and contributes to our understanding of the hypothesized “mite-bomb” and “robber-lure” mechanisms via which large numbers of *Varroa* may transmit from 1 collapsing colony to a neighboring healthy colony (Peck and Seeley 2019), especially as mites have been shown to facultatively switch host preferences to aid in dispersal at high densities (Cervo et al. 2014). We highlight that our results must be understood alongside the observation that the largest colonies may host the largest absolute number of parasites despite relatively low levels of per-capita parasitism, these colonies arguably pose a higher risk to their neighbors than smaller, allegedly “more-sick” colonies if they suddenly collapse, and will not necessarily be treated if beekeepers are following a strict IPM framework. Management decisions may need to ensure that fast-growing, large populations of *Varroa* are not exploiting large, apparently healthy colonies as refugia only to cause very high per-capita parasitism rates across an apiary once colonies transition to overwintering and substantially contract. Flexible thresholds for mite control are already used as part of industry recommendation based on time of year, for example, recommending immediate treatment if per-capita parasitism is > 1% for overwintering, compared to > 3% for population peak as stated in the Honey Bee Health Coalition’s “Tools for Varroa Management” (2022), but could be modified to scale to whole apiaries based on these results. Whether such control strategies would help slow down any putative selection for more virulent *Varroa* under certain ecological circumstances is worthy of future theoretical explorations.

In summary, we present a field experimental demonstration of a virulence × growth-rate relationship in a critical honey bee parasite, the *Varroa* mite. We show that this manifests at the scale of the apiary, where neighboring colonies pay the price of fast-growing *Varroa* populations in nearby colonies. This highlights the need to consider control efforts at the correct spatial scale, rather than at the scale of individual colonies, and foregrounds the role of spatial structure in determining the epidemiological outcomes of parasite ecology, evolution, and control.

## Supplementary Material

Supplementary material is available at *Journal of Insect Science* online.

ieae049_suppl_Supplementary_Figures_S1-S3

## Data Availability

All necessary data and analysis are available on a Zenodo-archived GitHub repository. DOI: 10.5281/zenodo.10899687.
