# Supplementary material for: Faster-growing parasites threaten host populations via patch-level population dynamics and higher virulence; a case study in Varroa mites (Mesostigmata: Varroidae) and honey bees (Hymenoptera: Apidae)
Source: J Insect Sci. 2024 May 28;24(3):17. doi: 10.1093/jisesa/ieae049 (PMC11132124; doi:10.1093/jisesa/ieae049)
Supplement: ieae049_suppl_Supplementary_Figures_S1-S3 [file ieae049_suppl_supplementary_figures_s1-s3.docx]

**Faster-growing parasites threaten host populations via patch-level population dynamics and higher virulence; a case study in *Varroa* mites (Mesostigmata: Varroidae) and honey bees (Hymenoptera: Apidae).**

Lewis J. Bartlett^1,5^*, Michael Boots^2,3^, Berry J. Brosi^4^, Keith S. Delaplane^5^, Travis L. Dynes^6^, Jacobus C. de Roode^7^

1 Center for the Ecology of Infectious Diseases, Odum School of Ecology, University of Georgia, GA USA
2 Department of Integrative Biology, UC Berkeley, CA USA

3 Biosciences, University of Exeter, Penryn Campus, UK.
4 Department of Biology, University of Washington, WA USA
5 Department of Entomology, University of Georgia, GA USA
6 Department of Environmental Sciences, Emory University, GA USA
7 Department of Biology, Emory University, GA USA
***lewis.bartlett@uga.edu**

*ORCID*LJB 0000-0002-4418-8071
MB 0000-0003-3763-6136
BJB 0000-0002-9233-1151
KSD 0000-0001-9323-441X
TLD 0000-0002-6533-6037
JCdR 0000-0002-8423-8918


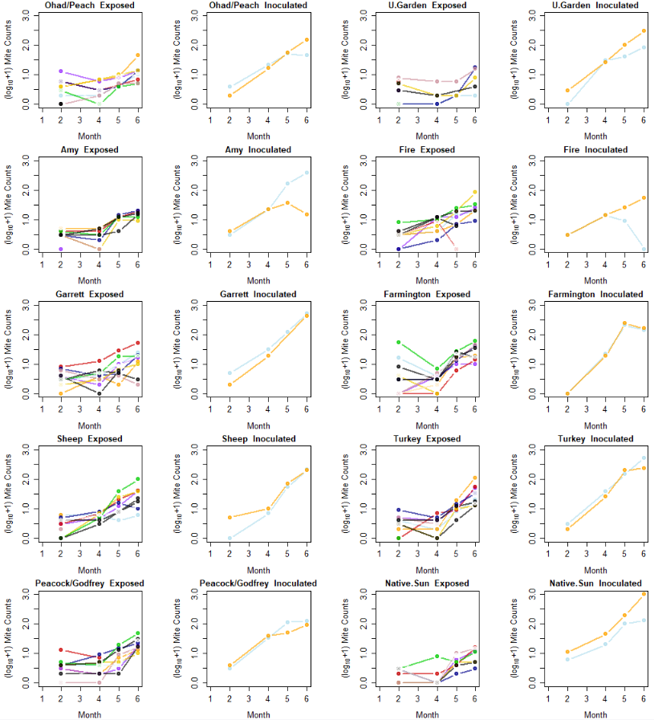
**Supplementary Figures - SI**

**Figure S1** – Mite population growth in each colony, split by apiary and whether colonies were inoculated with mites or exposed to mites in neighboring colonies and the landscape. Colors simply help distinguish each individual colony within a panel, and only correspond between panels in that the same colors are used for exposed or inoculated colonies. Estimates of mite population growth rates used in the analysis were taken from these 6-month data sets. Mite counts are transformed to allow for much easier graphical interpretability.


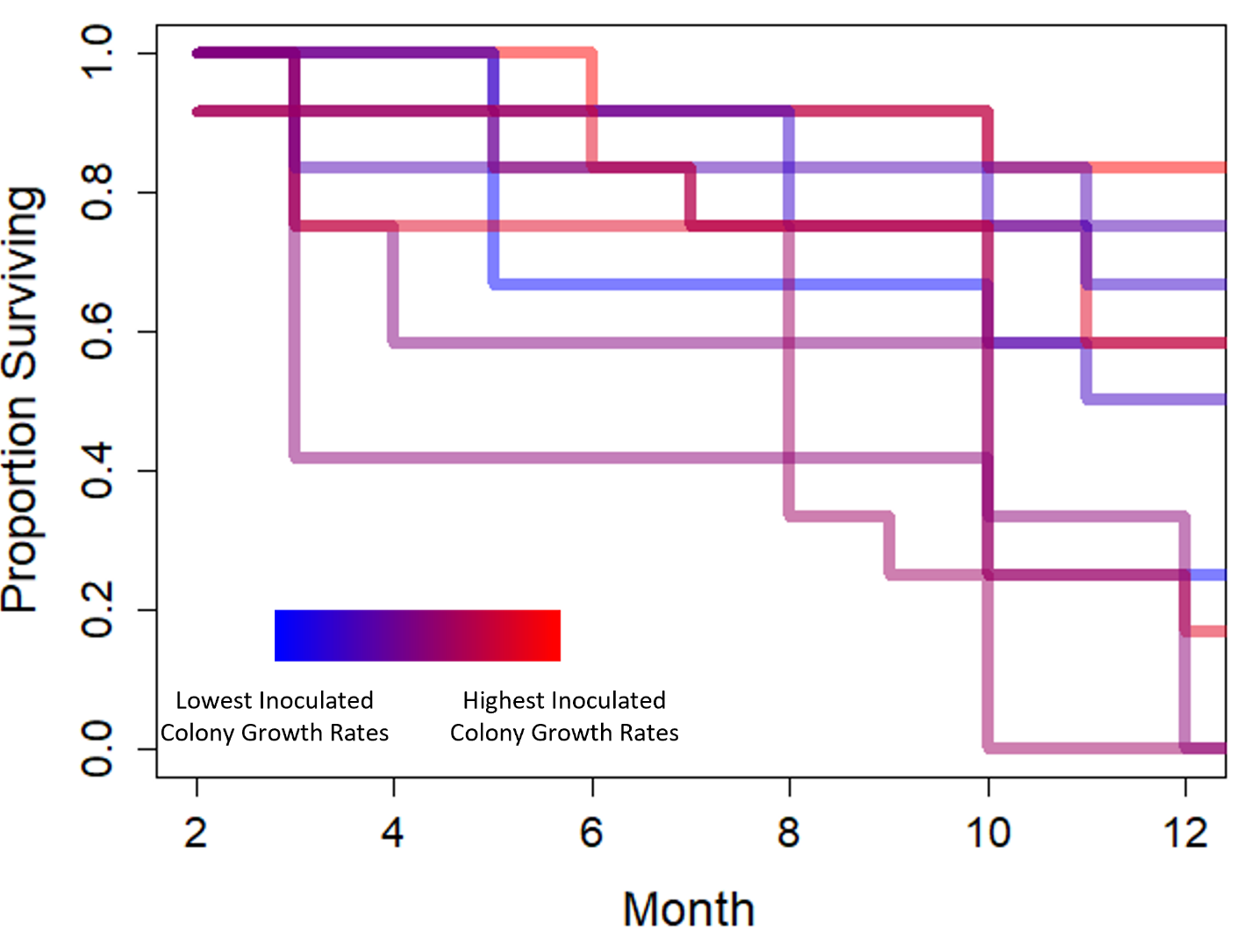


**Figure S2** – Survival analysis of **exposed colonies only** for each apiary across the 12 months of the observation period, corresponding to a full year from *Varroa* clearance and subsequent inoculation of two other colonies within each apiary. Each line corresponds to one apiary, with color shading denoting the mite population growth rate measured in the inoculated colonies placed in those apiaries (inoculated colonies are excluded from the survival analysis). Our survival analysis found a significant association between mite population growth in inoculated colonies and the survival of neighboring exposed colonies in that apiary, where faster growth rates corresponded to higher mortality rates (p = 0.030).


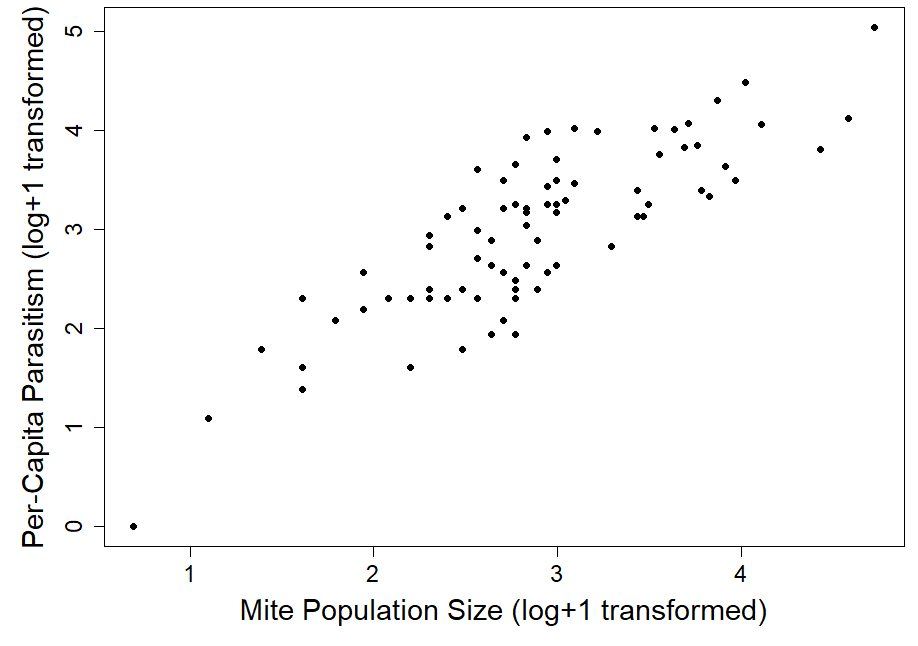


**Figure S3** – Correlation between mite population size (measured by sticky screens, x-axis) and per-capita mite parasitism (measured by alcohol washes, y-axis) as colonies enter overwintering (each point represents one exposed colony; inoculated colonies were excluded from this analysis). Total mite population size was strongly, significantly positively correlated with per-capita mite parasitism rates at the end of the season. Mite counts are transformed to allow for easier graphical interpretability.
